# Underrated Cause of Hypoxemia and Stroke in Patient with Cirrhosis

**DOI:** 10.5334/jbsr.1912

**Published:** 2019-10-09

**Authors:** Thibaut Lapotre, Colin Desir, Benoît Ghaye

**Affiliations:** 1 Cliniques Universitaires Saint Luc, BE; 2 CHU de Liège, BE

**Keywords:** hepatopulmonary syndrome, stroke, hypoxemia, CT scan, arteriovenous fistula

## Abstract

The subpleural arteriovenous shunts that are hallmarks of hepatopulmonary syndrome may cause stroke.

## Report

A 76-year-old-woman was admitted for right hemiparesis. Her medical history included cirrhosis with portal hypertension known for 14 years. Her heart rhythm was regular, with a heart rate of 65 beats per minute. Brain MRI demonstrated an acute ischemic lesion in the left part of the pons. A brain cortical atrophic sequela was also seen in the right occipital lobe. Vascular arteriosclerosis and any cardiac cause of stroke were excluded by dedicated imaging. During the neurological revalidation, she developed a platypnea. SpO2 was <90%. Clinical examination demonstrated diffuse spider naevi predominantly on the abdominal wall. Chest CT angiography ruled out pulmonary embolism. MIP reconstructions in lung window showed dilated pulmonary vessels abutting the pleura on the non-dependent part of the right lung (Figure A, black arrows). CT angiography volume-rendering (VR) reformat (Figure B) and cinematic VR reformat (Figure C) demonstrated subpleural arteriovenous shunts in the middle lobe (thin arrow) between enlarged pulmonary artery (arrowhead) and pulmonary vein (large arrow). These imaging findings strongly suggested hepatopulmonary syndrome (HPS). Hydrothorax, atelectasis and pneumonia were also excluded. Unfortunately, the patient quickly deteriorated and died a few days later.

**Figure A:**
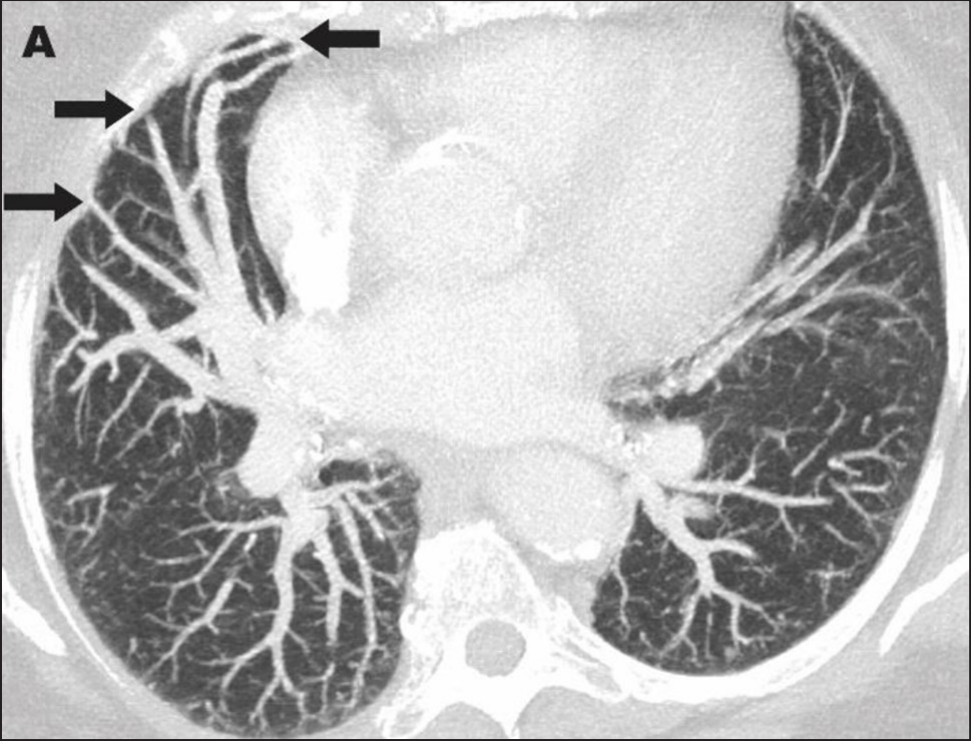


**Figure B:**
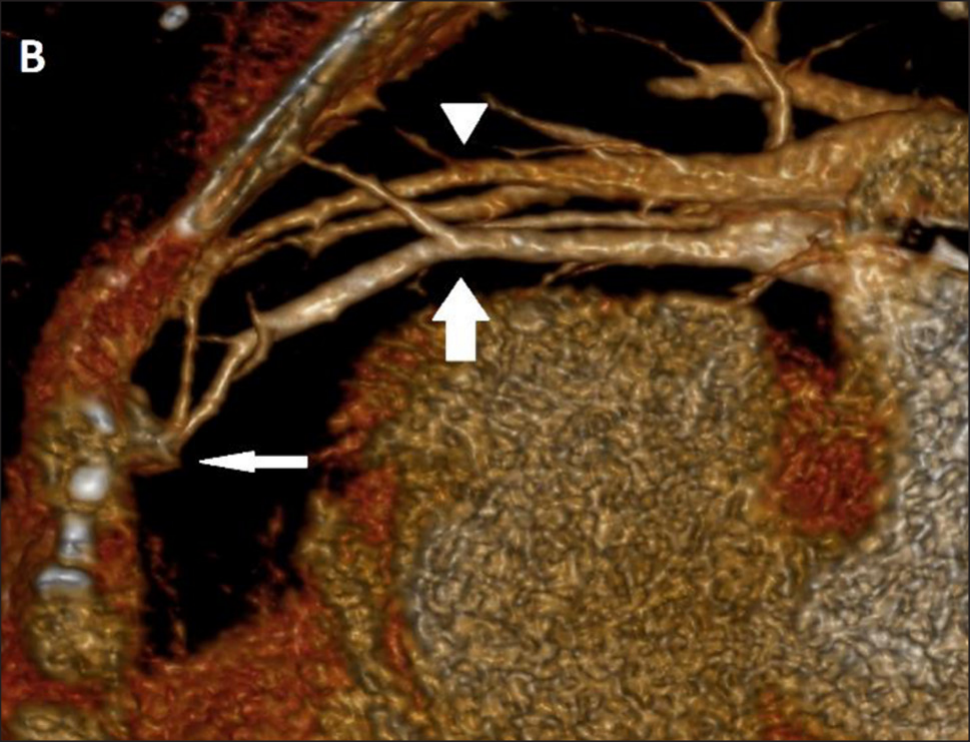


**Figure C:**
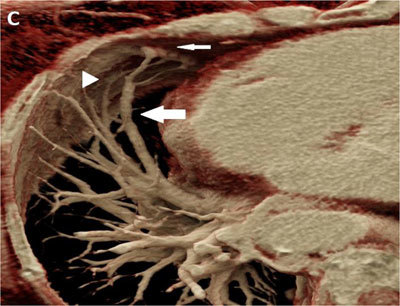


## Comment

HPS is defined by a triad including liver disease regardless of type and severity, oxygenation impairment and intrapulmonary vascular dilatation resulting in right-to-left shunting [1]. Contrast-echocardiography is currently preferred to 99 mTc-radiolabelled MAA (macro-aggregated albumin) to confirm intrapulmonary shunting [1]. CT shows diffuse and distal vascular dilatation even located in non-dependent-areas. It may sometimes also demonstrate focal arteriovenous malformation [1]. Excessive arteriovenous shunting can result in a poor response to 100% oxygen breathing [1]. Main HPS complications include hypoxemia proportional to the severity of these abnormalities and paradoxical embolization [1]. Brain stroke may be an occasional cause of death in patients with HPS [1]. A severe form of HPS (PaO2 < 60 mmHG), as opposed to severe portopulmonary hypertension, is an indication for liver transplantation [1].
